# Biological functions of casein kinase 1 isoforms and putative roles in tumorigenesis

**DOI:** 10.1186/1476-4598-13-231

**Published:** 2014-10-11

**Authors:** Birgit Schittek, Tobias Sinnberg

**Affiliations:** Department of Dermatology, Division of Dermatooncology, University of Tübingen, Liebermeisterstr. 25, 72076 Tübingen, Germany

**Keywords:** Casein kinase 1, Tumor, Cancer, Melanoma, β-catenin, p53

## Abstract

Isoforms of the casein kinase 1 (CK1) family have been shown to phosphorylate key regulatory molecules involved in cell cycle, transcription and translation, the structure of the cytoskeleton, cell-cell adhesion and receptor-coupled signal transduction. They regulate key signaling pathways known to be critically involved in tumor progression. Recent results point to an altered expression or activity of different CK1 isoforms in tumor cells. This review summarizes the expression and biological function of CK1 family members in normal and malignant cells and the evidence obtained so far about their role in tumorigenesis.

**Electronic supplementary material:**

The online version of this article (doi:10.1186/1476-4598-13-231) contains supplementary material, which is available to authorized users.

## Introduction

Protein kinases represent an important therapeutic target for drug development since they play a central role in signal transduction. By reversible phosphorylation of their substrate proteins, kinases exert influence on substrate activity, localization and function and thus play an essential role in almost all cellular processes. The casein kinases (CK) belong to the serine/threonine kinases and can be subdivided further into either casein kinase 1 (CK1) or casein kinase 2 (CK2) families due to their high homology in their catalytic domains [1]. This review focuses on the isoforms of the CK1 family, which have non-redundant and essential functions in cell survival and tumorigenesis [2].

**Figure 1:**
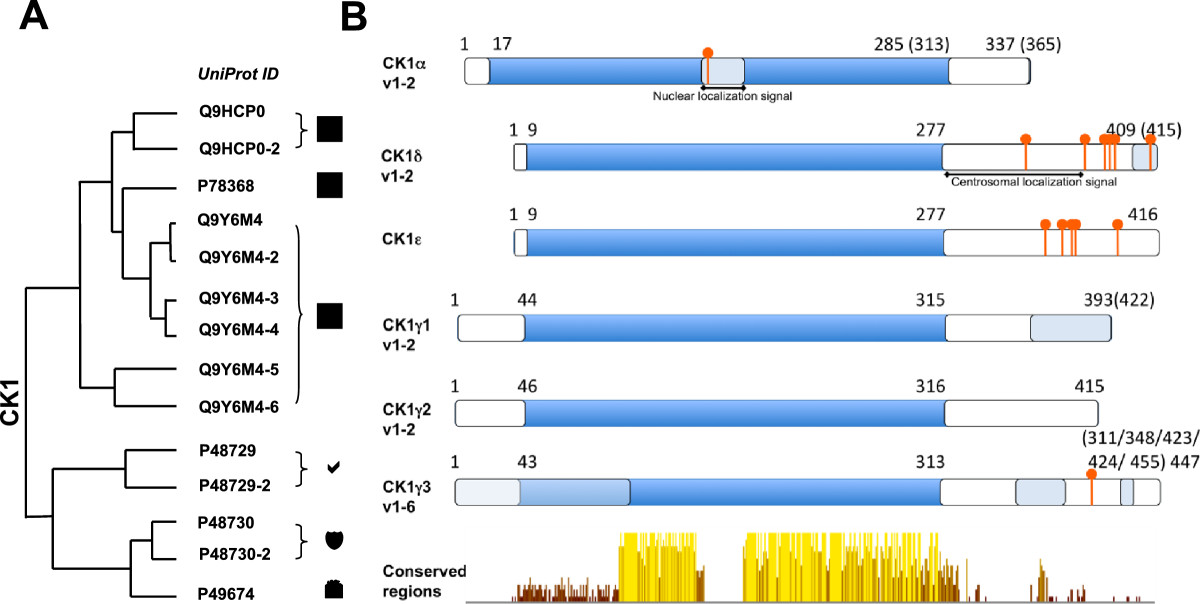
**Structure of casein kinase 1 family members. (A)** Homology tree of the casein kinase 1 family consisting of six human casein kinase 1 isoforms: alpha, delta, epsilon, gamma1, gamma2 and gamma3. All available and corresponding protein sequences were retrieved from UniProt (see IDs) and the aligned sequences were used to calculate the average distance tree (using the BLOSUM62 algorithm) visualized with jalview. **(B)** Schematic drawings of the isoforms show conserved regions (yellow color) especially within the kinase domains (dark blue). Variable regions due to transcript variants and alternative splicing are depicted in light blue resulting in variants differing in protein length: CK1α: 337/365 aa; CK1δ: 409/415 aa; CK1ϵ: 416 aa; CK1γ1 393/422 aa; CK1γ2: 415 aa; CK1γ3: 311–455 aa. Phosphorylation sites (red) are occurring predominantly at the C-terminal ends of the delta (Ser331/370/382/383/384/411) and epsilon isoforms (Ser343/354/362/363/389) and are known to be auto-inhibitory. An additional phosphorylation site can be found within the 28 aa insertion of CK1α (Ser156). A nuclear localization signal is located in the long variant of the alpha isoform (aa 160–163). A centrosomal localization signal [7] is located at the C-terminus in the delta (aa 278–364) isoform.

In vertebrates, seven CK1 isoforms (α, β, γ1, γ2, γ3, δ and ϵ) and several splice variants for CK1α, δ, ϵ and γ3 have been identified [1, 3]. The CK1β-isoform has only been found in cows. They differ in length and sequence of the N-terminal (9–76 amino acids) and especially the C-terminal (24–200 amino acids) non-catalytic domain (Figure 1). By this, the molecular weight of the various CK1 isoforms ranges from 37 kD (CK1α) up to 51 kD (CK1γ3). The C-terminal domain plays a crucial role in substrate specificity and in the regulation of kinase activity [1, 3–5]. The isoforms show a high homology, i.e. CK1δ and CK1ϵ are 98% identical in their kinase domain and 53% identical in their C-terminal regulatory domain [6]. Therefore, there is some redundancy with respect to substrate phosphorylation - however, there are also examples of distinct biological roles for the different CK1 isoforms.

### Substrates and interaction partners of CK1 isoforms

In recent years an increasing number of substrates have been identified that are phosphorylated by CK1 isoforms *in vitro* or *in vivo*[1, 4, 5]*.* Several known substrates especially of the CK1α and δ isoforms are involved in oncogenic signaling pathways as Wnt/β-catenin (β-catenin; dishevelled (DVL); adenomatous polyposis coli (APC); nuclear factor of activated T cells, cytoplasmic 3 (NFATC3)), p53 (p53; p53/E3 ubiquitin-protein ligase Mdm2 (MDM2)), PI3K/AKT (forkhead box protein O1 (Foxo1)), and death receptor signaling (Fas-associated death domain protein (FADD); BH3-interactive domain death agonist (BID)). In addition, various interaction partners have been identified from which it is not known yet whether they can serve as a CK1 substrate. These include proteins that are involved in cell cycle, apoptosis induction, DNA repair, mitochondrial function and signal transduction. Moreover, several proteins involved in oncogenic signaling pathways are predicted to interact with the different CK1 isoforms (Table 1). These proteins mainly belong to the Hedgehog (GLI), Hippo (MST, YAP), Wnt/β-catenin (Axin, Dvl1-3, FZD1 and 5, GSK3, Wnt3A), NFκB (NFκBIA), TGF-beta/Smad (Smad3) or p53 (MDM2 and 4) -signaling pathways and hence are involved in regulation of the cell cycle, apoptosis induction or cell survival. Many of these interactants are known to be deregulated in tumor cells and interaction with CK1 isoforms might trigger tumor initiation or progression [2].

**Table 1:** **CK1 interaction partners and substrates**

Protein	Description	CK1 interaction partners	Known Phospho-rylation sites	Cellular process	Reference
**BCL10**	B-cell CLL/lymphoma 10	α		Apoptosis	[8]
**BID**	BH3 interacting domain death agonist	α, δ	T59, S64 (α, δ)	Apoptosis	[9]
**FADD**	Fas (TNFRSF6)-associated via death domain	α	S194 (α)	Apoptosis	[10]
**E2F1**	E2F transcription factor 1	α		Cell cycle	[11]
**Chk1**	Checkpoint kinase 1	δ		Cell cycle	[12]
**DDX3**	DEAD box RNA helicase	ϵ		DNA repair	[13]
**SPRY2**	Sprouty1	δ, ϵ		FGF-signaling	[14]
**GLI1-3**	GLI family zinc finger 1-3	α, δ, ϵ, γ1, γ2, γ3		Hedgehog signaling	[15]
**ANT2**	Adenine nucleotide translocase 2	ϵ		Mitochondrial function	[16]
**MST1**	Mammalian sterile 20-like kinase 1	ϵ		Hippo signaling	[17]
**NFKBIA**	Nuclear factor of kappa light polypeptide gene enhancer in B-cells inhibitor, alpha	α		NFκB signaling	[18]
**P65**	NFκB subunit	γ1	S536	NFκB signaling	[19]
**MDM2 MDMX (MDM4)**	Mouse double minute 2 or 4 homolog	α		p53 signaling	[20]
**TP53**	Tumor protein p53	α, δ, ϵ, γ1, γ2, γ3	S6, S9 (α); T18 (δ)	p53 signaling	[21]
**Foxo1**	Forkhead box O1	δ, ϵ, γ1, γ2, γ3	S322 (α, γ1), S325 (α)	PI3K/AKT signaling	[22]
**RAPGEF2**	Rap guanine nucleotide ex-change factor 2	α	S1244, S1248	Ras activation	[23]
**SMAD3**	SMAD family member 3	γ2, ϵ		TGF-beta/Smad signaling	[24]
**APC**	Adenomatous polyposis coli	α, δ, ϵ	all ϵ: S1279, S1392, S1504, S1505, S1507, S1510	Wnt signaling	[25]
**AXIN1 AXIN2**	Axin 1 and 2	α, δ, ϵ, γ1		Wnt signaling	[4]
**CTNNB1**	Beta-catenin	α, δ, ϵ	S45 (α, δ)	Wnt signaling	[4]
**NFATC3**	Nuclear factor of activated T-cells	α	all α: T204, S207, T210, S211, S215	Wnt signaling	[26]
**DVL1-3**	Dishevelled, dsh homolog 1-3	δ, ϵ, γ1		Wnt signaling	[4]
**CSNK1D**	Casein kinase 1, delta	ϵ		Wnt signaling	[4]
**FZD1 FZD5**	Frizzled homolog 1 or 5	γ1		Wnt signaling	[27]
**GSK3A GSK3B**	Glycogen synthase kinase 3 alpha or beta	γ1		Wnt signaling	[28]
**WNT3A**	Wingless-type MMTV integration site family, member 3A	γ1		Wnt signaling	[29]

CK1 substrates are shown with known phosphorylation sites together with the involved cellular processes of the corresponding proteins. The following programs were used for determination of interaction or phosphorylation: http://string-db.org, http://hprd.org; http://regphos.mbc.nctu.edu.tw, http://www.phosphosite.org. Only proteins validated by biochemical assays are shown.

A distinctive feature of CK1 family members is their exclusive need of ATP to phosphorylate their substrates and their independency of other co-factors. They show a strong preference for primed, pre-phosphorylated substrates [30]. Surprisingly, the substrate specificity defined *in vitro* differs from *in vivo* for the different CK1 family members [4] suggesting that the *in vivo* specificity is regulated by interaction partners, autophosphorylation or subcellular localization. Interaction with cellular proteins has been shown to be a major determinant of the localization of CK1 isoforms [31–35] and to either enhance or inhibit their activity [12, 13, 36].

### Biological functions of CK1-isoforms

The wide range of substrates shows that the CK1 family members are involved in multiple cellular processes. For example they are involved in the regulation of membrane trafficking, cytokinesis, vesicular transport, ribosome biogenesis, DNA repair, signal transduction pathways and in the circadian rhythm [1, 5, 37]. Up to now most evidence points to important regulatory roles of the isoforms CK1α, CK1δ and CK1ϵ, while the role of the gamma-isoforms are still enigmatic and not very well investigated.

*CK1α* plays a role in the mitotic spindle formation during cell division and in DNA repair mechanisms and participates in RNA metabolism [1]. Antibodies specific for CK1α block cell cycle progression during M phase in mouse oocytes, which indicates that CK1α is required for proper cell cycle progression in these cells [38, 39]. CK1α can be found at the centrosomes, microtubule asters and the kinetochore [40]. In addition, it was shown that mTOR cooperates with CK1α to promote its own full activation via the sustained degradation of the endogenous mTOR inhibitor DEPTOR [41]. Similarly, CK1α regulates apoptotic signaling pathways, however, there seems to be cell type-specific differences. CK1α has been shown to have an anti-apoptotic function in the extrinsic apoptosis pathway. Its inhibition increased Fas-induced apoptosis in Hela cells, whereas the overexpression of CK1α delayed cell death, caused by the phosphorylation of BID, which prevented the caspase 8 dependent cleavage of BID [9]. In addition, CK1α inhibits TRAIL induced apoptosis by modification of the TNF receptor or FADD at the death-inducing signaling complex (DISC) [42]. Therefore downregulation of CK1α leads to an enhancement of TRAIL-induced cell death. Likewise, CK1α promotes cell survival by interacting with the retinoid X receptor (RXR). Downregulation of CK1α enhances the apoptotic effect of RXR agonists [43]. In contrast, overexpression of CK1α in metastatic melanoma cells induces apoptosis [44].

In addition to the regulatory function in apoptosis signaling pathways, CK1α is involved in the phosphorylation of G-protein coupled receptors (GPCRs) such as the M3 and M1 muscarinic receptors and rhodopsin [45]. These become phosphorylated by CK1α upon agonist-induced desensitization [45, 46]. Furthermore, CK1α is involved in the phosphorylation of NFAT4 (nuclear factor for activated T cells 4), so that its nuclear localization signal is masked and thus the activity of the transcription factor is inhibited [47, 48]. Recently, it was shown in epithelial cells that in response to factors promoting cell motility, CK1α in conjunction with IKKβ phosphorylate the Rap guanine exchange factor 2 (RAPGEF2) leading to its degradation by the proteasome which was shown to be essential for the invasion of breast cancer cells [23].

*CK1δ and CK1ϵ* are known to be important regulators in the circadian rhythm of eukaryotic cells. The central clock genes period circadian protein homolog 1–3 (PER1-3) cycle in a daily rhythm. Cytoplasmic PER is phosphorylated by CK1, which induces its degradation [49]. When PER is in a complex with cryptochrome (CRY), the phosphorylation site is masked and the complex together with CK1 translocates to the nucleus where it represses another clock transcription factor complex (the BMAL1/Clock complex) and inhibits the transcription of most of the clock genes. Additionally, CK1α was identified as a negative PER1 regulator similar to the CK1δ isoform [50], however the binding of CK1δ and ϵ to PER proteins seems to be much stronger than CK1α [50]. Pharmaceutical inhibition of CK1ϵ was less effective compared to pan CK1δ/ϵ inhibitors in prolonging the circadian rhythm [51] proposing a dominant role of CK1δ in the regulation of daily oscillating processes. Taken together CK1s are important regulators of the circadian rhythm [52, 53]. Meanwhile it is known that CK1δ is involved in the regulation of cell cycle progression and it was recently shown that checkpoint kinase 1 (Chk1) is able to interact and specifically phosphorylates CK1δ and by this regulates the kinase activity [12]. Additionally, CK1δ interacts with the spindle apparatus and regulates phosphorylation of α−, β−, and γ − tubulin [35, 54, 55]. It seems to be essential for microtubule nucleation at the golgi [56]. Besides tubulin phosphorylation CK1δ mediates the phosphorylation of microtubule associated proteins (MAPs), stathmin and tau thereby regulating the dynamics of the microtubule and spindle apparatus [34, 35, 57–59]. Recently, it was shown that CK1δ mediates the phosphorylation of Sid4 an important cytokinesis regulator [60] strengthening the important function of CK1δ in cell proliferation. As CK1δ and Chk1 are known to inhibit p53 via phosphorylation, pharmacological inhibition of both kinases resulted in activation of p53 similar to the effect of the MDM2 inhibitor nutlin-3 [61]. CK1δ and CK1ϵ are able to interact with Sprouty2 (SPRY2), which is a potent negative regulator of fibroblast growth factor (FGF) receptor tyrosine kinase signaling. CK1 activity and binding are necessary for SPRY2 mediated inhibition of FGF-stimulated neurite outgrowth and FGF-dependent RAS activation via GRB2/SOS [14]. It was further shown that CK1δ and CK1ϵ are required for the biogenesis of small ribosomal subunits and for the functionality of 40S ribosomal subunits in protein translation [37].

### The role of CK1 isoforms in Wnt/β-catenin signaling

The CK1 isoforms CK1α, CK1δ and CK1ϵ have a common regulatory function in the Wnt/β-catenin signaling pathway and act in a concerted manner [2]. Dishevelled (Dvl), a key component in the Wnt/β-catenin signaling pathway, becomes phosphorylated by CK1δ/ϵ upon pathway activation by Wnts [62]. Recent evidence indicates that there is a coordinated action of the different CK1 isoforms to activate Wnt/β-catenin signaling in colon adenocarcinoma cells [29]. In these cells CK1ϵ, but not CK1α is required for the early response to Wnt3a stimulation. The two protein kinases function sequentially: CK1ϵ is bound to p120-catenin and E-cadherin and is required for early responses to Wnt3a stimulation, such as recruitment of Dishevelled 2 (Dvl-2), followed by the phosphorylation of the Wnt co-receptors LRP5/6 by CK1γ which leads to the recruitment of axin complexed with CK1α. CK1α then phosphorylates p120-catenin and E-cadherin causing a release of p120-catenin/E-cadherin from the complex, and dissociation of CK1ϵ from p120 which terminates the input signal. For pathway inactivation β-catenin has to become N-terminally phosphorylated. In order to be phosphorylated by GSK3β at the Ser residues 33, 37 and 41, β-catenin must first get primed by phosphorylation on Ser45 by CK1α [63, 64]. Without priming phosphorylation by CK1α, GSK3β does not phosphorylate β-catenin and therefore β-catenin is not degraded. A down-regulation of CK1α thus leads to an accumulation of cytoplasmic β-catenin [44]. In mouse embryonic stem cells DNA damage and its response (DDR) causes an activation of the canonical Wnt signaling by reduced CK1α expression levels. The Wnt signaling was shown to limit the p53 dependent apoptosis induction after DDR indicating a pro-survival effect of Wnt signaling [65]. Recently, the DEAD-box RNA helicase DDX3 was identified as a regulator of the Wnt/β-catenin signaling pathway by acting as a regulatory subunit of CK1ϵ in a Wnt-dependent manner. It binds to CK1ϵ, directly stimulates its kinase activity, and promotes phosphorylation of the scaffold protein dishevelled [13]. Furthermore CK1ϵ is known to phosphorylate adenomatosis polyposis coli (APC) together with GSK3β which leads to an increased affinity of APC to β-catenin causing a transfer of β-catenin from axin to APC. This exposes β-catenin to the β-TrCP ubiquitin ligase marking it for its proteasomal degradation [25, 66]. Additionally CK1δ is capable to regulate the canonical Wnt signaling on the level of lymphoid enhanced binding factor 1 (Lef-1). Lef-1 is phosphorylated by CK1δ resulting in the disassembly of the β-catenin/Lef-1 transcription factor complex thereby inhibiting the pathway [67].

### The role of CK1 isoforms in p53 signaling

The tumor suppressor protein p53 as well as the p53 interacting proteins MDM2 and MDMX - both capable to regulate p53 activity by inhibitory interaction - are substrates of CK1 isoforms. The three CK1 isoforms CK1α, CK1δ and CK1ϵ are capable to N-terminally phosphorylate the p53 *in vitro* and *in vivo* resulting in activation of p53. Furthermore, CK1δ can phosphorylate MDM2, which leads to a reduced interaction with p53 and thus to stabilization and activation of p53 [68–70]. These data suggest that increased expression or activation of CK1α or CK1δ are able to activate p53 and by this increase cell cycle arrest and apoptosis induction. However, there are differences in the ability of the CK1 isoforms to either activate [70] or inactivate p53 [68, 69] activity depending on the cell types used or the experimental conditions. Knockdown or downregulation of CK1α in the intestinal epithelium of mice [71], in human colon cancers [72] or in leukemia cells [73] triggers p53 activation. Similarly, one study showed that CK1α stably associates with MDMX, stimulates MDMX-p53 binding, and cooperates with MDMX to inactivate p53 [20]. These data suggest that inhibition of CK1α activity increases p53 activity. Indeed, knockdown of CK1α induces p53 transcriptional activity by reducing the inhibitory effect of MDMX for p53 [74] since MDMX phosphorylation is necessary for interaction with p53. Furthermore, knockdown of CK1α reduces the interaction of MDM2 with p53 thus increasing p53 activity [11].

Under stress conditions and when CK1 is highly expressed the situation differs: p53 is activated by an autoregulatory loop between p53 and either CK1α or CK1δ. CK1α stimulates p53 probably by direct phosphorylation of Thr18 and Ser20 [5, 75]. Thereby, CK1α could be a cellular fine-tuning tool for the regulation of p53 activity. It was shown that after genotoxic stress it comes to a transcriptional activation of CK1δ by p53 [68]. Furthermore, DNA damage activates p53 in part by disrupting CK1α-MDMX interaction and reducing MDMX-p53 binding affinity [20]. In addition, DNA damage leads to an enhanced interaction between CK1δ/ϵ and MDM2 resulting in multisite phosphorylation of MDM2 by CK1 and enhanced MDM2 degradation and subsequently enhanced p53 activity. Inactivation of CK1 (primarily CK1δ or ϵ and to a lesser extent CK1α) results in accumulation of MDM2, thus decreased p53 activity and resistance to apoptosis induced by DNA damaging agents [76]. In conclusion, depending on the cell system used, CK1α and CK1δ are able to either increase or decrease p53 activity by direct phosphorylation or modulating the activity of the p53 interacting proteins MDM2 and MDMX. Furthermore, the activity of p53 as well as CK1α and CK1δ is increased under stress conditions pointing to an autoregulatory loop between CK1 isoforms and p53.

### Regulation of the expression and activity of CK1 isoforms

The expression and activity of CK1 family members seems to be tightly regulated. Protein expression levels can be found at http://www.proteinatlas.org. Surprisingly, not much is known concerning the mechanisms involved in regulating CK1 expression and kinase activity until now. The expression of CK1α can be down-regulated by the *miRNA* miR-155 [77]. *Treatment* of cells with insulin, topoisomerase inhibitors, irradiation or viral transformation is able to increase CK1 activity [1]. The subcellular localization of the CK1 isoforms is mainly regulated by binding to intracellular structures or protein complexes and thereby plays a role in substrate-recognition and CK1 activity [32–35, 37, 55]. Studies on the circadian rhythm in mice showed that the nuclear localization of CK1δ and CK1ϵ is regulated during the day [78].

*Posttranscriptional mechanisms* are involved in the regulation of CK1α activity. In Hela cells two CK1α transcripts have been described which result from alternative splicing [79, 80]. The short 2.4kB transcript originates from the larger 4.2kB transcript due to the use of an alternative polyadenylation site. Interestingly, the longer transcript has six adenine uridine rich element (ARE) motifs in the 3'-untranslated region (in the short transcript there is only one ARE-motif), which lead to increased RNA degradation of the 4.2 kB transcript. Moreover, the long transcript harbors the above mentioned insertion of 84 bp or 28 amino acids in the catalytic domain, which also causes a change in substrate affinity [79]. Only the long splice variants of CK1α have a nuclear localization sequence (PVGKRKR), which is responsible for the transport of CK1α into the nucleus [81].

The activity of CK1 isoforms can also be regulated by *post-translational modifications*, in particular phosphorylations or proteolytic processing. For CK1δ and CK1ϵ it was shown that the C-terminal domain is autophosphorylated, which inhibits the kinase activity [82–84]. As mentioned earlier, CK1ϵ is activated after Wnt3a stimulation by the removal of inhibitory C-terminal phosphorylations. The responsible phosphatases are not identified yet. Furthermore, part of the C-terminal domain can be cleaved, which increases kinase activity [1]. In addition, the *three dimensional structure* of CK1δ plays a role in the regulation of activity. CK1δ forms dimers leading to an inhibition of kinase activity [85].

*Allosteric regulatory mechanisms* were recently identified by two working groups and seem to be major determinants of CK1 kinase activity. Cruciat *et al.* showed that ATP-dependent RNA helicase DDX3X (DDX3) binds to CK1ϵ thereby allosterically activating the kinase domain and triggering canonical Wnt signaling. Using biochemical methods this novelty in kinase regulation was extended to the isoforms alpha, delta and gamma2 [13]. It is estimated that different DDX proteins can allosterically activate these CK1 isoforms (up to five fold). In line with these findings was the identification of the CK1α activating drug pyrvinium. This anthelmintic can bind to all CK1 isoforms but activates only CK1α due to conformational changes, thereby strongly inhibiting canonical Wnt signaling [86].

### CK1 Isoform expression and tumorigenesis

**Figure 2:**
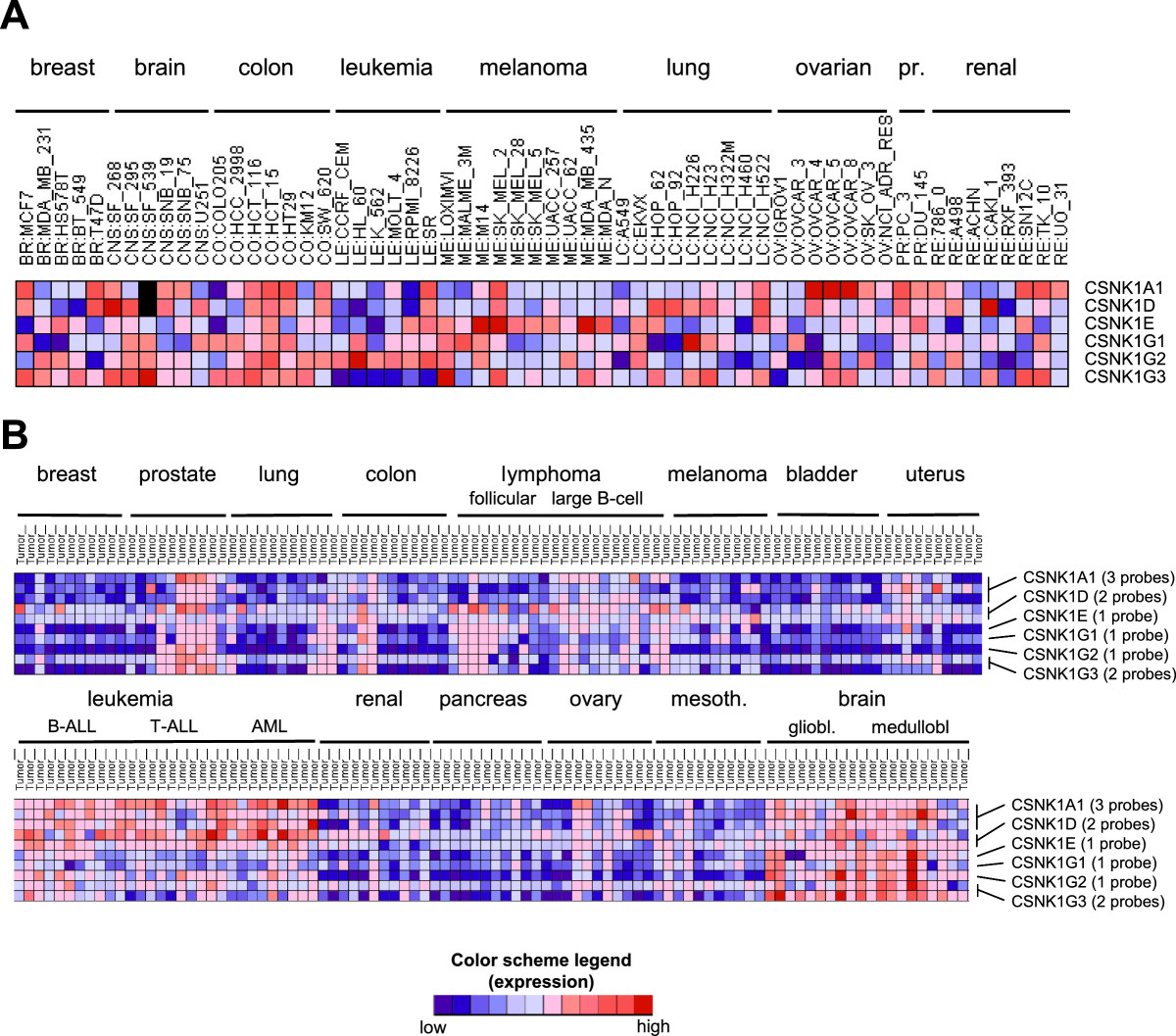
**Expression of CK1 isoforms in cancer. (A)** Shown are the color-coded RNA expression levels of CK1α, δ, ϵ, γ1, γ2, ϵ3 (CSNK1A1, CSNK1D, CSNK1E, CSNK1G1, CSNK1G2, CSNK1G3) in different tumor types as determined by the CellMiner database for gene expression analysis of 60 cancer cell lines from the NCI60 panel. Z-scores for the six isoforms were calculated and subjected to the Gene-Pattern analysis software for heatmap generation using the Gene-Pattern online tools (http://genepattern.broadinstitute.org/gp/pages/index.jsf). Blue depicts low expression levels and red high expression levels. **(B)** The Ramaswamy Multi-cancer dataset (public available at http://www.broadinstitute.org/cancer/software/genepattern/datasets/) was used for analysis of the z-scored expression levels in different tumor tissue samples and again used for heatmap illustration.

The evidence obtained in recent years emphasizes the importance of CK1 isoforms in cancer progression in different types of tumors [2]. Microarray database analyses from tumor cell lines (http://discover.nci.nih.gov/cellminer/analysis.do) and tissues (http://www.broadinstitute.org/cancer/software/genepattern/datasets/;RamaswamyMulti-cancer) indicated that the CK1δ and CK1ϵ isoforms are overexpressed on RNA level in many tumor types. This includes cancers of bladder, brain, breast, colorectal, kidney, lung, melanoma, ovarian, pancreatic, prostate and the hematopoetic system. Expression of the CK1γ1-3 isoforms seems to be rather low in different cancers types (Figure 2A, B). In addition, it has been shown that CK1δ is overexpressed in cells of hyperplastic B cell follicles and B cell lymphomas in p53 deficient mice [87]. In renal cell carcinoma increased CK1γ3 expression was found [88]. In choriocarcinoma an increased expression of CK1δ [55] and in ductal adenocarcinomas of the pancreas an increased expression of CK1δ and CK1ϵ was detected [89]. A tabulary overview can be found in the recent review of Knippschild et al. [90].

RNA expression of CK1α is more variable with high expression in cancers of the brain, prostate, lymphoma and especially in leukemia (Figure 2A, B). In bladder cancer, lung cancer and melanoma there is a trend to decreased RNA expression of CK1α (Figure 2A, B). Expression analysis of CK1α in melanoma datasets of the oncomine.org expression database clearly revealed a reduction in mRNA expression during melanoma progression. We confirmed the reduction of CK1α expression on protein level [44]. The downregulation of CK1α expression in progressed melanoma tumors seems to be mainly mediated by promoter methylation of the CK1α gene [44]. In contrast, there is one recent report claiming that CK1α expression is higher in melanoma compared to benign nevi [91].

**Figure 3:**
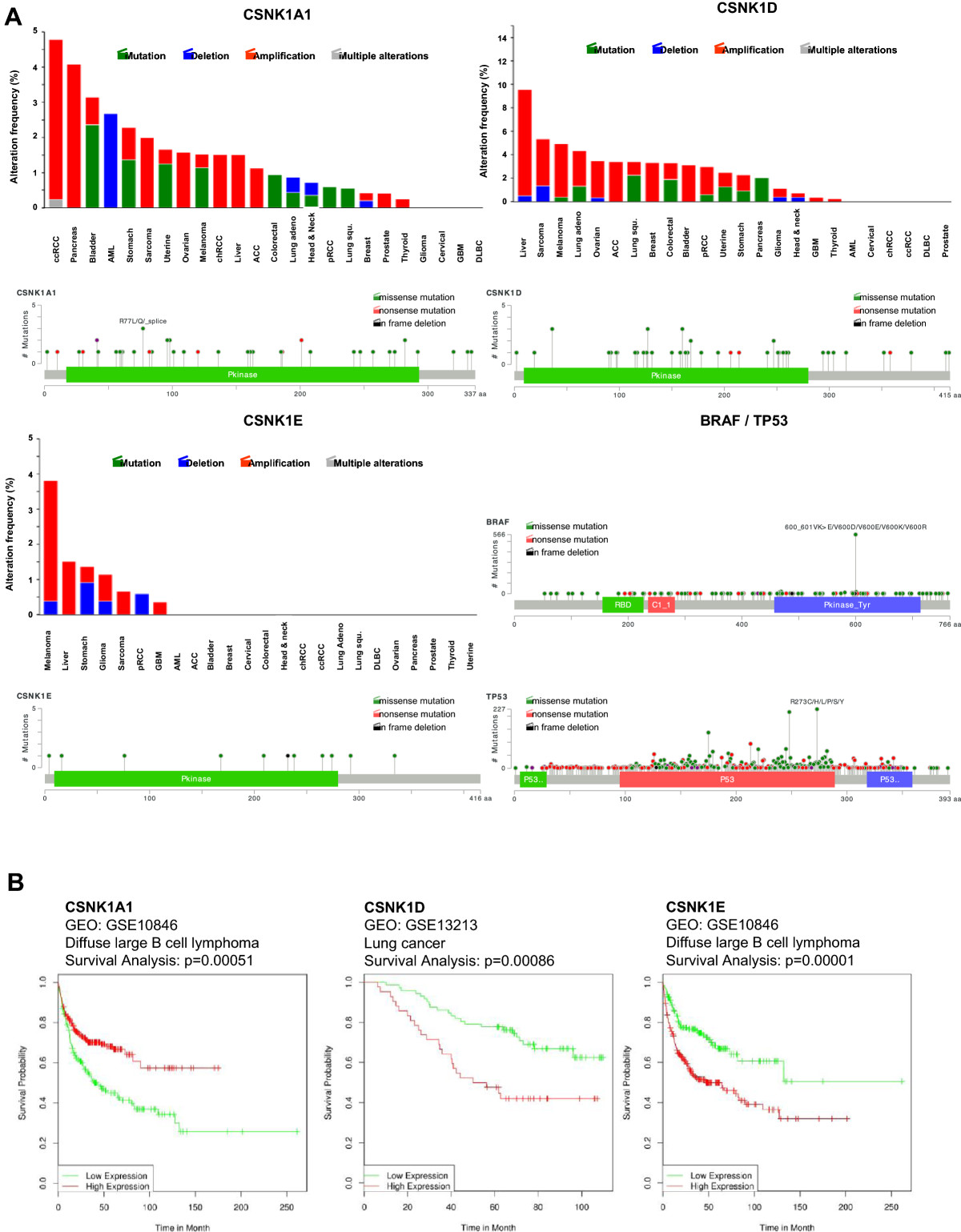
**Mutations in CK1 isoforms and cancer patient survival. (A)** Mutation analysis of CK1 isoforms and overall survival of tumor patients stratified in CK1 isoform expression classes. 24 different tumor entities were analyzed using the cBioPortal for Cancer Genomics [94, 95] accessing the newest TCGA datasets. The highest mutation frequency for CK1α was detected in clear cell renal cell carcinoma (ccRCC) with approx. 4.8%, for CK1δ in liver cancer with approx. 9.5% and for CK1ϵ with approximately 3.8% (top diagrams). The distribution along the primary structure of the three CK1 isoforms α, δ and ϵ was analyzed in the cBioportal using all available datasets (69 cancer studies containing data of 17584 tumor samples). No hotspot mutation could be identified and only very low mutation frequencies were found (bottom diagrams). In comparison, analysis of the oncogene BRAF and the tumor suppressor TP53 revealed high mutation frequencies and the hot spot mutations at BRAFV600D/E/K/R or TP53R273C/H/L/P/S/Y. **(B)** Survival analysis of stratified cancer patients into high and low CK1 isoform expression (determined by microarray gene expression analysis) was performed using the PPISURV online tool [96]. The studies showing the highest significant differences in overall survival (OS) between CK1high and CK1low patients are shown as Kaplan Meier curves for the α, δ and ϵ isoforms (CK1 high expression in red, CK1 low expression in green).

The expression data suggests that there are tumor-cell specific differences in the functional activities and/or relevance of CK1 isoforms in cancer. However, to gain information about the relevance of the different CK1 isoforms in tumor progression, the CK1 protein expression as well as the kinase activity should be determined since it is known that CK1 activity is regulated by posttranscriptional mechanisms and posttranslational modifications (see above). Several CK1- substrates (see Table 1) are known to play a role in tumorigenesis and are substrates of different CK1-isoforms. Therefore it will be important to clarify the redundancy in the activity of different CK1 isoforms. Furthermore, the mechanisms regulating CK1 expression and activity in tumor cells are still not well understood. Despite promoter methylation [44] mutations could play a regulatory role. In breast carcinoma, mutations were found in the coding region of the CK1ϵ gene [92]. In colonic adenoma, R324H mutations are described in the CK1δ gene, and these were correlated with an increased oncogenic potential of the lesions including a higher transformation potential *in vitro*[93]. Interestingly, the R324H mutation did not significantly alter the CK1δ kinase activity or the Wnt/β-catenin signaling activity *in vitro* but had strong impact on the morphological movement like secondary axis formation in *Xenopus* embryos. Mutations in CK1 isoforms seems to be rare, however, copy number variations seem to be more frequent (see http://cancer.sanger.ac.uk/cosmic). Analysis of the actual datasets of the cancer genome atlas (TCGA) available at the cBioPortal for Cancer Genomics [94, 95] revealed the mutation pattern and copy number alterations (CNA) in 24 different tumor types (Figure 3). The total alteration frequency including CNAs and mutations is generally rather low ranging from 4.8% in clear cell renal cell carcinoma (ccRCC) for CSNK1A1 over 9.5% in liver cancer for CSNK1D to 3.8% for CSNK1E in melanoma tumors. Specific point mutation frequencies are even lower and do not exceed 2.4% (as for CSNK1A1 in bladder cancer, for CSNK1D in lung squamous carcinoma and colorectal or pancreatic cancer; point mutations in CSNK1E seem to be very rare). Moreover the distribution of the detected mutations along the primary structure of the CK1 isoforms shows no accumulation in certain domains or regions which excludes the occurrence of hotspot mutations like in BRAF or TP53 (Figure 3A). Therefore genomic alterations of CK1 isoforms which lead to functional hyperactivity or defects may occur, however they seem to be seldom events.

### Functional role of CK1 isoforms in tumor progression

What are the functional consequences of deregulated CK1 expression in tumor cells? On a functional level it was reported that CK1ϵ enhances the β-catenin-dependent proliferation in breast cancer [97]. However, it was recently shown that patients with oral cancers who had a loss of CK1ϵ expression had a poorer overall survival rate than patients who expressed CK1ϵ [98]. For CK1δ it was shown that a point mutation in the C-terminal regulatory domain of CK1δ (R342H) promotes the emergence of colorectal adenomas independent of Wnt-/β-catenin [93]. In contrast, a downregulation of CK1δ and ϵ-isoforms induced cell cycle arrest and apoptosis in a variety of tumor cell lines of different origin. These effects were also Wnt/β-catenin-independent, but dependent on activated RAS and inactive p53 [5, 99]. It was also shown that impaired CK1δ activity attenuates SV40-induced cellular transformation *in vitro* and mouse mammary carcinogenesis *in vivo*[100]. Thus, depending on the experimental setup and the tumor type CK1-isoforms seems to have a specific and non-redundant role in tumorigenesis, however the results are often contradictory. One reason might be that CK1 isoforms have also a regulatory function, which is independent of the kinase activity. It was described that CK1ϵ can interact with mitochondrial proteins in ovarian cancer cells and by this, increase growth and survival of the tumor cells in a Wnt/β-catenin independent manner [16].

Until now most hints pointing to an important role in tumor progression have been described for CK1α. Our own previous work support the observation that CK1α in comparison to the other CK1 isoforms have a dominant and non-redundant function in metastatic melanoma cells [44]. We identified CK1α as a novel tumor suppressor and a key regulator of β-catenin signaling in melanoma cells. Knockdown of CK1α in primary melanoma cell lines significantly enhanced the invasive capacity of melanoma cell lines *in vitro*. This pro-invasive effect after CK1α knockdown was reversible by inhibition of β-catenin signaling, demonstrating the dependence of the invasive phenotype on β-catenin in melanoma cells. Moreover, knockdown of CK1α in a melanoma xenograft model using melanoma cells with low intrinsic tumorigenic potential strongly increased tumorigenicity and stabilized β-catenin [101]. In line with, one study showed that a pharmacological increase of CK1α protein level and thereby a destabilization of activated β-catenin significantly diminished melanoma cell migration [102]. Interestingly Chien et al. found high β-catenin levels to be rather a good prognostic marker in melanoma as well as to inhibit invasion probably by the promotion of differentiation in conjunction with MITF [103, 104]. In another recent study Chien et al. described a bad overall survival of BRAF inhibitor treated patients with tumors expressing high β-catenin protein levels. This reveals that the different results probably refer to different, so far non-identified melanoma subtypes [105].

Furthermore, the activation of CK1α kinase by the above mentioned allosteric activator pyrvinium induces cell death in colon carcinoma cell lines [86]. However, pyrvinium was recently described to elicit cytotoxicity in a CK1 independent manner by AKT downregulation and GSK3 activation in 293 T cells [28] and it is known to inhibit the unfolded protein response by GRP78 suppression [106] as well as cancer cell protective autophagy [107]. During melanoma progression CK1α expression is lost and is not necessary for survival and cell cycle progression in metastatic melanoma cells. Overexpression of CK1α retarded proliferation of metastatic melanoma cells and induced cell death, whereas the primary melanoma cell lines were mostly unaffected in their cell growth. This is consistent with the data showing that re-expression of CK1α in the lung cancer cell line A549 in which the expression of CK1α is also low causes reduced cell proliferation *in vitro* and tumor growth *in vivo*[108]. Our results suggest that the effect of CK1α on proliferation and invasion of melanoma cells in different stages of growth is at least partly due to an effect on the Wnt/β-catenin signaling pathway [44, 101].

The effect of CK1α on tumor progression seems to be also dependent on the activity of the tumor suppressor p53. One study shows that loss of CK1α in the intestinal epithelium of mice leads to hyperproliferation but not to tumor development. An additional inactivation of the p53 tumor suppressor gene or its target p21 is necessary to ensure the emergence of high-invasive colorectal tumors [71]. This suggests a non-redundant and dominant role of CK1α compared to the other CK1 isoforms not only in melanoma, but also in colorectal tumorigenesis. Moreover expression levels of CK1α seem to be a predictive survival marker in colon cancer patients with non-functional p53. Low CK1α expression levels were shown to correlate with poor survival rates and a significant hazard ratio of 4.7 hinting at a pro-oncogenic function of loss-of-CK1α on a p53 deficient background [72]. Furthermore, CK1α knockdown as well as additional inactivation of p53 is necessary for tumors to progress. If p53 is present, tumor cells will be eliminated by cell cycle arrest or apoptosis. This assumption was verified recently in acute myeloid leukemia, where it was shown that inhibition of CK1α has only a therapeutic effect, when p53 is active [73].

In the concept of tumorigenesis oncogenes play a pivotal role. However, excessive proliferative signaling as well as sustained DNA damage response can trigger cell senescence, commonly associated with a senescence-associated secretory phenotype (SASP). The secreted proteins include proinflammatory chemokines and tissue remodeling factors that have on the one hand an autocrine, senescence reinforcing effect and on the other hand a paracrine effect on neighbouring cells, leading to bystander senescence and immune cell recruitment. Interestingly, in cancer, SASP can increase proliferation and invasion of tumor cells or induce angiogenesis. Another recently described pattern of secreted proteins from senescent cancer cells can be subsumed as senescent-inflammatory response (SIR) resulting in a cell-autonomous initiation of inflammation, causing colonic tumor progression in a p21 deficient background. Usually, knockdown of CK1α induces p53/p21 mediated arrest and a senescent phenotype in p53 functional cells [71]. The group of Ben-Neriah found out that in p53 (or p21) deficient murine gut tumor cells the SIR factors that occur after CK1α knockdown induce pro-invasive genes like PROX1 thereby causing highly aggressive and hyperproliferative tumor cells. The described effect was highly dependent on the inflammatory response itself [109]. This shows that CK1α is a negative regulator of the SIR and loss of CK1α causes oncogene-like chronic stress which induces a pro-oncogenic switch to cell-autonomous, tumor promoting inflammation when p53 is inactivated.

In order to show the impact of CK1 isoforms on tumor progression we analyzed the survival of different cancer patients (17 types of cancer) depending on the gene expression levels of the three CK1 isoforms α, δ and ϵ using the PPISURV database [96], This database includes 45 expression studies with approximately 8000 patient survival data sets. Analysis revealed after stratification of the patients into the two classes high and low CK1α expressing tumors a significant correlation between prolonged overall survival (OS) and high expression levels for 12 (B cell lymphoma, breast cancer, leukemia and lung cancer) out of 17 studies, whereas in 5 studies (lung cancer, colon cancer and liposarcoma) patients with high expression levels did worse. For CK1δ there was positive correlation between high expression and longer survival in 6 (breast cancer, leukemia and glioma) out of 9 studies (3 correlated negatively) and for CK1ϵ there was a rather mixed picture with 4 positive (glioma, lung cancer and leukemia) and 4 negative correlating studies (B cell lymphoma, breast cancer and lung cancer) (Table 2 and Figure 3B). The higher number of significant studies showing a positive prognosis for patients with high levels of CK1α expression hints to the postulated function as tumor suppressor in some cancers whereas the mixed pattern for CK1δ and especially CK1ϵ depicts a context dependent function of the isoforms being either pro- or anti-tumorigenic.

**Table 2:** **Survival data from the PPISURV database showing correlations of CK1 gene expression and overall survival (OS) of cancer patients**

Survival profile of CSNK1A1 across available datasets with (with p < 0.05)				
**GEO dataset**	**Cancer type**	**GENE (Probe ID)**	**P-value**	**Effect sign**
GSE10846	Diffuse large b cell lymphoma	206562_S_AT	0.00051	**Positive**
GSE25065	Breast cancer	208865_AT	0.000669	**Positive**
GSE25055	Breast cancer	206562_S_AT	0.00141	**Positive**
GSE11121	Breast cancer	208867_S_AT	0.00174	**Positive**
GSE39671	Chronic lymphocytic leukemia	226920_AT	0.0021	**Positive**
GSE7390	Breast cancer	208867_S_AT	0.00576	**Positive**
GSE13213	Lung cancer	A_24_P251899	0.00715	**Negative**
GSE24080	Multiple myeloma	208865_AT	0.00736	**Positive**
GSE22762	Chronic lymphocytic leukemia	208866_AT	0.0119	**Positive**
GSE2034	Breast cancer	206562_S_AT	0.0167	**Positive**
GSE31210	Lung cancer	208866_AT	0.0179	**Positive**
GSE3494	Breast cancer	208866_AT	0.0256	**Positive**
GSE17536	Colon cancer	1556006_S_AT	0.0271	**Negative**
GSE11969	Lung cancer	21556	0.0284	**Negative**
GSE17538	Colon cancer	1556006_S_AT	0.0289	**Negative**
GSE30682	Breast cancer	ILMN_1775058	0.033	**Positive**
GSE30929	Liposarcoma	208865_AT	0.0461	**Negative**
**Survival profile of CSNK1D across available datasets (with p < 0.05)**				
**GEO dataset**	**Cancer type**	**GENE (Probe ID)**	**P-value**	**Effect sign**
GSE131213	Lung cancer	A_23_P207896	0.000863	**Negative**
GSE25025	Breast cancer	208774_AT	0.00101	**Positive**
GSE39671	Chronic lymphocytic leukemia	207945_S_AT	0.00382	**Positive**
GSE22226	Breast cancer	41429	0.00432	**Positive**
GSE22762	Chronic lymphocytic leukemia	208774_AT	0.0122	**Positive**
GSE31210	Lung cancer	207945_S_AT	0.0162	**Negative**
GSE25065	Breast cancer	208774_AT	0.023	**Positive**
GSE18166	Astrocytic gliomas	37202	0.0268	**Positive**
GSE13041	Glioblastoma	208774_AT	0.0272	**Negative**
**Survival profile of CSNK1E across available datasets (with p < 0.05)**				
**GEO dataset**	**Cancer type**	**GENE (Probe ID)**	**P-value**	**Effect sign**
GSE10846	Diffuse large b cell lymphoma	226858_AT	1.39e-05	**Negative**
GSE1456	Breast cancer	222015_AT	0.002	**Negative**
GSE11969	Lung cancer	13708	0.016	**Negative**
GSE4271	High-grade glioma	202332_AT	0.0281	**Positive**
GSE24450	Breast cancer	ILMN_1708858	0.0296	**Negative**
GSE18166	Astrocytic gliomas	28635	0.0303	**Positive**
GSE31210	Lung cancer	202332_AT	0.032	**Positive**
GSE39671	Chronic lymphocytic leukemia	234943_AT	0.0478	**Positive**

However, it remains uncertain whether the CK1δ and ϵ isoforms represent drivers of tumorigenesis or if their disregulation and altered expression is rather the consequence of other cellular oncogenic events. Therefore critical genetic validation models should be developed in order to prove the concept of CK1 isoforms as important regulators in tumorigenesis besides the development of specific pharmacological inhibitors.

### Pharmacological inhibition of CK1 isoforms

Due to the above described involvement of CK1 isoforms in tumorigenesis pharmacological inhibition of CK1 familiy members could be of interest for targeted cancer therapy. However, the development of isoform specific inhibitors seems to be a demanding task since the first known inhibitors like the ATP-competitive type I inhibitors CKI-7 or IC261 are not highly specific and show a rather broad effectivity against CK1 isoforms with a predominance for δ and ϵ. IC261 showed activity against pancreatic tumors in a xenograft mouse model [89] as well as in a MYC-driven neuroblastoma model [110]. Meanwhile, it is known that IC261 also efficiently inhibits the polymerization of microtubules in a CK1 independent manner, Therefore it is questionable whether the anti-tumorigenic effects are due to selective inhibition of CK1δ. D4476, another CK1 inhibitor which shows some selectivity towards the δ isoform, was successfully applied in a leukemia mouse model causing the elimination of the tumor cells. However, since the α isoform seems to be dominant for the described mechanism in this model by Jaras et al., the effects measured with D4476 might result due to the inhibition of additional susceptible isoforms and drug targets like CK1δ or the TGF-β type-I receptor ALK5. Huart et. al reported a novel inhibitor belonging to the class of pyrazolo-pyridine analogues that strongly inhibits CK1δ in conjunction with CHK1 thereby activating the p53 pathway and inducing apoptosis in A375 melanoma cells. However, a similar analog described in the same study but lacking the CHK1 inhibitory function showed no apoptosis induction in A375 cells [61]. Further developments were undertaken by Bibian et al. in order to generate highly specific CK1δ/ϵ inhibitors resulting in a series of purine scaffold inhibitors (SR-1277, SR-2890, SR-3029) with IC_50_ values in the low nanomolar range. These inhibitors were also highly potent in inhibiting A375 melanoma cell growth in a MTT viability assay. Interestingly, the inhibition with less specific inhibitors like D4476 or PF670462 did not show these inhibitory effects, proposing a better cell penetration of the SR inhibitors and better efficacy [111]. In order to exclude any of the remaining very few off-targets to be the main originators of the effects on A375 melanoma cell viability, genetic tests (like knockdowns or knockouts) targeting CK1 isoforms should be performed. Further studies will be needed to determine the cellular efficacy and specificity of these interesting novel CK1 inhibitors. For detailed further information about the development of additional CK1 inhibitors we refer to the excellent review by Knippschild et al. [90].

## Conclusions

CK1 isoforms can influence the development and progression of tumor cells, although they seem to have different effects depending on the tumor types. Their ability to regulate several important signaling molecules is modulated in different types of tumors. This suggests that CK1 isoforms might be suitable targets for clinical intervention. Especially their ability to regulate p53 and Wnt-signaling, cell cycle progression and apoptosis induction makes them attractive targets in tumor therapy. Since the CK1 isoforms seems to have sometimes opposing roles in different tumor types it will be essential in future to validate the effect of specific CK1 isoforms in defined tumor types on cell cycle progression and signal transduction. Since several kinase inhibitors are in pre-clinical development there is hope that, among these, some of the most effective inhibitors could delay or inhibit tumor progression.

## Authors’ original submitted files for images

Below are the links to the authors’ original submitted files for images. Authors’ original file for figure 1 Authors’ original file for figure 2 Authors’ original file for figure 3

## References

[1] Knippschild U, Gocht A, Wolff S, Huber N, Löhler J, Stöter M (2005). The casein kinase 1 family: participation in multiple cellular processes in eukaryotes. Cell Signal.

[2] Knippschild U, Wolff S, Giamas G, Brockschmidt C, Wittau M, Wörl PU, Eismann T, Stöter M, Würl PU, Stöter M (2005). The role of the casein kinase 1 (CK1) family in different signaling pathways linked to cancer development. Onkologie.

[3] Gross SD, Anderson RA (1998). Casein kinase I: spatial organization and positioning of a multifunctional protein kinase family. Cell Signal.

[4] Price MA (2006). CKI, there’s more than one: casein kinase I family members in Wnt and Hedgehog signaling. Genes Dev.

[5] Cheong JK, Virshup DM (2011). Casein kinase 1: Complexity in the family. Int J Biochem Cell Biol.

[6] Fish KJ, Cegielska A, Getman ME, Landes GM, Virshup DM (1995). Isolation and characterization of human casein kinase I epsilon (CKI), a novel member of the CKI gene family. J Biol Chem.

[7] Greer YE, Rubin JS (2011). Casein kinase 1 delta functions at the centrosome to mediate Wnt-3a-dependent neurite outgrowth. J Cell Biol.

[8] Carvalho G, Le Guelte A, Demian C, Vazquez A, Gavard J, Bidère N (2010). Interplay between BCL10, MALT1 and IkappaBalpha during T-cell-receptor-mediated NFkappaB activation. J Cell Sci.

[9] Desagher S, Osen-Sand A, Montessuit S, Magnenat E, Vilbois F, Hochmann A, Journot L, Antonsson B, Martinou JC (2001). Phosphorylation of bid by casein kinases I and II regulates its cleavage by caspase 8. Mol Cell.

[10] Alappat EC, Feig C, Boyerinas B, Volkland J, Samuels M, Murmann AE, Thorburn A, Kidd VJ, Slaughter CA, Osborn SL, Winoto A, Tang W-J, Peter ME (2005). Phosphorylation of FADD at serine 194 by CKIalpha regulates its nonapoptotic activities. Mol Cell.

[11] Huart A-S, MacLaine NJ, Meek DW, Hupp TR (2009). CK1alpha plays a central role in mediating MDM2 control of p53 and E2F-1 protein stability. J Biol Chem.

[12] Bischof J, Randoll S-J, Süßner N, Henne-Bruns D, Pinna LA, Knippschild U (2013). CK1δ kinase activity is modulated by Chk1-mediated phosphorylation. PLoS One.

[13] Cruciat C-M, Dolde C, de Groot REA, Ohkawara B, Reinhard C, Korswagen HC, Niehrs C (2013). RNA helicase DDX3 is a regulatory subunit of casein kinase 1 in Wnt-β-catenin signaling. Science.

[14] Yim DGR, Ghosh S, Guy GR, Virshup DM (2014). Casein kinase 1 regulates Sprouty2 in FGF-ERK signaling. Oncogene.

[15] Price MA, Kalderon D (2002). Proteolysis of the Hedgehog signaling effector Cubitus interruptus requires phosphorylation by Glycogen Synthase Kinase 3 and Casein Kinase 1. Cell.

[16] Rodriguez N, Yang J, Hasselblatt K, Liu S, Zhou Y, Rauh-Hain JA, Ng S-K, Choi P-W, Fong W-P, Agar NYR, Welch WR, Berkowitz RS, Ng S-W (2012). Casein kinase I epsilon interacts with mitochondrial proteins for the growth and survival of human ovarian cancer cells. EMBO Mol Med.

[17] Xu F, Wang Y-L, Chang J-J, Du S-C, Diao L, Jiang N, Wang H-J, Ma D, Zhang J (2014). Mammalian sterile 20-like kinase 1/2 inhibits the Wnt/β-catenin signalling pathway by directly binding casein kinase 1ϵ. Biochem J.

[18] Bidère N, Ngo VN, Lee J, Collins C, Zheng L, Wan F, Davis RE, Lenz G, Anderson DE, Arnoult D, Vazquez A, Sakai K, Zhang J, Meng Z, Veenstra TD, Staudt LM, Lenardo MJ (2009). Casein kinase 1alpha governs antigen-receptor-induced NF-kappaB activation and human lymphoma cell survival. Nature.

[19] Wang Y, Hu L, Tong X, Ye X (2014). Casein kinase 1γ1 inhibits the RIG-I/TLR signaling pathway through phosphorylating p65 and promoting its degradation. J Immunol.

[20] Wu S, Chen L, Becker A, Schonbrunn E, Chen J (2012). Casein kinase 1α regulates an MDMX intramolecular interaction to stimulate p53 binding. Mol Cell Biol.

[21] Milne DM, Palmer RH, Campbell DG, Meek DW (1992). Phosphorylation of the p53 tumour-suppressor protein at three N-terminal sites by a novel casein kinase I-like enzyme. Oncogene.

[22] Rena G, Bain J, Elliott M, Cohen P (2004). D4476, a cell-permeant inhibitor of CK1, suppresses the site-specific phosphorylation and nuclear exclusion of FOXO1a. EMBO Rep.

[23] Magliozzi R, Low TY, Weijts BGMW, Cheng T, Spanjaard E, Mohammed S, van Veen A, Ovaa H, de Rooij J, Zwartkruis FJT, Bos JL, de Bruin A, Heck AJR, Guardavaccaro D (2013). Control of epithelial cell migration and invasion by the IKKβ- and CK1α-mediated degradation of RAPGEF2. Dev Cell.

[24] Waddell DS, Liberati NT, Guo X, Frederick JP, Wang X-F (2004). Casein kinase Iepsilon plays a functional role in the transforming growth factor-beta signaling pathway. J Biol Chem.

[25] Rubinfeld B, Tice DA, Polakis P (2001). Axin-dependent phosphorylation of the adenomatous polyposis coli protein mediated by casein kinase 1epsilon. J Biol Chem.

[26] Shen T, Liu Y, Cseresnyés Z, Hawkins A, Randall WR, Schneider MF (2006). Activity- and calcineurin-independent nuclear shuttling of NFATc1, but not NFATc3, in adult skeletal muscle fibers. Mol Biol Cell.

[27] Klein TJ, Jenny A, Djiane A, Mlodzik M (2006). CKIepsilon/discs overgrown promotes both Wnt-Fz/beta-catenin and Fz/PCP signaling in Drosophila. Curr Biol.

[28] Venerando A, Girardi C, Ruzzene M, Pinna LA (2013). Pyrvinium pamoate does not activate protein kinase CK1, but promotes Akt/PKB down-regulation and GSK3 activation. Biochem J.

[29] Del Valle-Pérez B, Arqués O, Vinyoles M, de Herreros AG, Duñach M (2011). Coordinated action of CK1 isoforms in canonical Wnt signaling. Mol Cell Biol.

[30] Flotow H, Graves PR, Wang AQ, Fiol CJ, Roeske RW, Roach PJ (1990). Phosphate groups as substrate determinants for casein kinase I action. J Biol Chem.

[31] McKenzie JAG, Riento K, Ridley AJ (2006). Casein kinase I epsilon associates with and phosphorylates the tight junction protein occludin. FEBS Lett.

[32] Sillibourne JE, Milne DM, Takahashi M, Ono Y, Meek DW (2002). Centrosomal anchoring of the protein kinase CK1delta mediated by attachment to the large, coiled-coil scaffolding protein CG-NAP/AKAP450. J Mol Biol.

[33] Wolff S, Stöter M, Giamas G, Piesche M, Henne-Bruns D, Banting G, Knippschild U (2006). Casein kinase 1 delta (CK1delta) interacts with the SNARE associated protein snapin. FEBS Lett.

[34] Wolff S, Xiao Z, Wittau M, Süssner N, Stöter M, Knippschild U (2005). Interaction of casein kinase 1 delta (CK1 delta) with the light chain LC2 of microtubule associated protein 1A (MAP1A). Biochim Biophys Acta.

[35] Behrend L, Stöter M, Kurth M, Rutter G, Heukeshoven J, Deppert W, Knippschild U (2000). Interaction of casein kinase 1 delta (CK1delta) with post-Golgi structures, microtubules and the spindle apparatus. Eur J Cell Biol.

[36] Yin H, Laguna KA, Li G, Kuret J (2006). Dysbindin structural homologue CK1BP is an isoform-selective binding partner of human casein kinase-1. Biochemistry.

[37] Zemp I, Wandrey F, Rao S, Ashiono C, Wyler E, Montellese C, Kutay U (2014). CK1δ and CK1ϵ are components of human 40S subunit precursors required for cytoplasmic 40S maturation. J Cell Sci.

[38] Brockman JL, Gross SD, Sussman MR, Anderson RA (1992). Cell cycle-dependent localization of casein kinase I to mitotic spindles. Proc Natl Acad Sci U S A.

[39] Gross SD, Simerly C, Schatten G, Anderson RA (1997). A casein kinase I isoform is required for proper cell cycle progression in the fertilized mouse oocyte. J Cell Sci.

[40] Wang L, Lu A, Zhou H-X, Sun R, Zhao J, Zhou C-J, Shen J-P, Wu S-N, Liang C-G (2013). Casein kinase 1 alpha regulates chromosome congression and separation during mouse oocyte meiotic maturation and early embryo development. PLoS One.

[41] Duan S, Skaar JR, Kuchay S, Toschi A, Kanarek N, Ben-Neriah Y, Pagano M (2011). mTOR generates an auto-amplification loop by triggering the βTrCP- and CK1α-dependent degradation of DEPTOR. Mol Cell.

[42] Izeradjene K, Douglas L, Delaney AB, Houghton JA (2004). Casein kinase I attenuates tumor necrosis factor-related apoptosis-inducing ligand-induced apoptosis by regulating the recruitment of fas-associated death domain and procaspase-8 to the death-inducing signaling complex. Cancer Res.

[43] Zhao Y, Qin S, Atangan LI, Molina Y, Okawa Y, Arpawong HT, Ghosn C, Xiao J-H, Vuligonda V, Brown G, Chandraratna RAS (2004). Casein kinase 1alpha interacts with retinoid X receptor and interferes with agonist-induced apoptosis. J Biol Chem.

[44] Sinnberg T, Menzel M, Kaesler S, Biedermann T, Sauer B, Nahnsen S, Schwarz M, Garbe C, Schittek B (2010). Suppression of casein kinase 1alpha in melanoma cells induces a switch in beta-catenin signaling to promote metastasis. Cancer Res.

[45] Tobin AB (2002). Are we beta-ARKing up the wrong tree? Casein kinase 1 alpha provides an additional pathway for GPCR phosphorylation. Trends Pharmacol Sci.

[46] Waugh MG, Challiss RA, Berstein G, Nahorski SR, Tobin AB (1999). Agonist-induced desensitization and phosphorylation of m1-muscarinic receptors. Biochem J.

[47] Okamura H, Garcia-Rodriguez C, Martinson H, Qin J, Virshup DM, Rao A (2004). A conserved docking motif for CK1 binding controls the nuclear localization of NFAT1. Mol Cell Biol.

[48] Dejmek J, Säfholm A, Kamp Nielsen C, Andersson T, Leandersson K (2006). Wnt-5a/Ca2 + −induced NFAT activity is counteracted by Wnt-5a/Yes-Cdc42-casein kinase 1alpha signaling in human mammary epithelial cells. Mol Cell Biol.

[49] Vielhaber E, Eide E, Rivers A, Gao ZH, Virshup DM (2000). Nuclear entry of the circadian regulator mPER1 is controlled by mammalian casein kinase I epsilon. Mol Cell Biol.

[50] Hirota T, Lee JW, Lewis WG, Zhang EE, Breton G, Liu X, Garcia M, Peters EC, Etchegaray J-P, Traver D, Schultz PG, Kay SA (2010). High-throughput chemical screen identifies a novel potent modulator of cellular circadian rhythms and reveals CKIα as a clock regulatory kinase. PLoS Biol.

[51] Walton KM, Fisher K, Rubitski D, Marconi M, Meng Q-J, Sládek M, Adams J, Bass M, Chandrasekaran R, Butler T, Griffor M, Rajamohan F, Serpa M, Chen Y, Claffey M, Hastings M, Loudon A, Maywood E, Ohren J, Doran A, Wager TT (2009). Selective inhibition of casein kinase 1 epsilon minimally alters circadian clock period. J Pharmacol Exp Ther.

[52] Harms E, Young MW, Saez L (2003). CK1 and GSK3 in the Drosophila and mammalian circadian clock. Novartis Found Symp.

[53] Meng Q-J, Maywood ES, Bechtold DA, Lu W-Q, Li J, Gibbs JE, Dupré SM, Chesham JE, Rajamohan F, Knafels J, Sneed B, Zawadzke LE, Ohren JF, Walton KM, Wager TT, Hastings MH, Loudon ASI (2010). Entrainment of disrupted circadian behavior through inhibition of casein kinase 1 (CK1) enzymes. Proc Natl Acad Sci U S A.

[54] Behrend L, Milne DM, Stöter M, Deppert W, Campbell LE, Meek DW, Knippschild U (2000). IC261, a specific inhibitor of the protein kinases casein kinase 1-delta and -epsilon, triggers the mitotic checkpoint and induces p53-dependent postmitotic effects. Oncogene.

[55] Stöter M, Bamberger AM, Aslan B, Kurth M, Speidel D, Löning T, Frank HG, Kaufmann P, Löhler J, HenneBruns D, Deppert W, Knippschild U (2005). Inhibition of casein kinase I delta alters mitotic spindle formation and induces apoptosis in trophoblast cells. Oncogene.

[56] Greer YE, Westlake CJ, Gao B, Bharti K, Shiba Y, Xavier CP, Pazour GJ, Yang Y, Rubin JS (2014). Casein kinase 1δ functions at the centrosome and Golgi to promote ciliogenesis. Mol Biol Cell.

[57] Hanger DP, Byers HL, Wray S, Leung K-Y, Saxton MJ, Seereeram A, Reynolds CH, Ward MA, Anderton BH (2007). Novel phosphorylation sites in tau from Alzheimer brain support a role for casein kinase 1 in disease pathogenesis. J Biol Chem.

[58] León-Espinosa G, García E, García-Escudero V, Hernández F, Defelipe J, Avila J (2013). Changes in tau phosphorylation in hibernating rodents. J Neurosci Res.

[59] Li G, Yin H, Kuret J (2004). Casein kinase 1 delta phosphorylates tau and disrupts its binding to microtubules. J Biol Chem.

[60] Johnson AE, Chen J-S, Gould KL (2013). CK1 is required for a mitotic checkpoint that delays cytokinesis. Curr Biol.

[61] Huart A-S, Saxty B, Merritt A, Nekulova M, Lewis S, Huang Y, Vojtesek B, Kettleborough C, Hupp TR (2013). A Casein kinase 1/Checkpoint kinase 1 pyrazolo-pyridine protein kinase inhibitor as novel activator of the p53 pathway. Bioorg Med Chem Lett.

[62] Bernatik O, Ganji RS, Dijksterhuis JP, Konik P, Cervenka I, Polonio T, Krejci P, Schulte G, Bryja V (2011). Sequential activation and inactivation of Dishevelled in the Wnt/beta-catenin pathway by casein kinases. J Biol Chem.

[63] Liu C, Li Y, Semenov M, Han C, Baeg GH, Tan Y, Zhang Z, Lin X, He X (2002). Control of beta-catenin phosphorylation/degradation by a dual-kinase mechanism. Cell.

[64] Amit S, Hatzubai A, Birman Y, Andersen JS, Ben-Shushan E, Mann M, Ben-Neriah Y, Alkalay I (2002). Axin-mediated CKI phosphorylation of beta-catenin at Ser 45: a molecular switch for the Wnt pathway. Genes Dev.

[65] Carreras Puigvert J, Von Stechow L, Siddappa R, Pines A, Bahjat M, Haazen LCJM, Olsen JV, Vrieling H, Meerman JHN, Mullenders LHF, Van De Water B, Danen EHJ (2013). Systems biology approach identifies the kinase Csnk1a1 as a regulator of the DNA damage response in embryonic stem cells. Sci Signal.

[66] Ha N-C, Tonozuka T, Stamos JL, Choi H-J, Weis WI (2004). Mechanism of phosphorylation-dependent binding of APC to beta-catenin and its role in beta-catenin degradation. Mol Cell.

[67] Hämmerlein A, Weiske J, Huber O (2005). A second protein kinase CK1-mediated step negatively regulates Wnt signalling by disrupting the lymphocyte enhancer factor-1/beta-catenin complex. Cell Mol Life Sci.

[68] Knippschild U, Milne DM, Campbell LE, DeMaggio AJ, Christenson E, Hoekstra MF, Meek DW (1997). p53 is phosphorylated in vitro and in vivo by the delta and epsilon isoforms of casein kinase 1 and enhances the level of casein kinase 1 delta in response to topoisomerase-directed drugs. Oncogene.

[69] Dumaz N, Milne DM, Meek DW (1999). Protein kinase CK1 is a p53-threonine 18 kinase which requires prior phosphorylation of serine 15. FEBS Lett.

[70] Winter M, Milne D, Dias S, Kulikov R, Knippschild U, Blattner C, Meek D (2004). Protein kinase CK1delta phosphorylates key sites in the acidic domain of murine double-minute clone 2 protein (MDM2) that regulate p53 turnover. Biochemistry.

[71] Elyada E, Pribluda A, Goldstein RE, Morgenstern Y, Brachya G, Cojocaru G, Snir-Alkalay I, Burstain I, Haffner-Krausz R, Jung S, Wiener Z, Alitalo K, Oren M, Pikarsky E, Ben-Neriah Y (2011). CKIα ablation highlights a critical role for p53 in invasiveness control. Nature.

[72] Sarasqueta AF, Forte G, Corver WE, de Miranda NF, Ruano D, van Eijk R, Oosting J, Tollenaar RAEM, van Wezel T, Morreau H (2013). Integral analysis of p53 and its value as prognostic factor in sporadic colon cancer. BMC Cancer.

[73] Järås M, Miller PG, Chu LP, Puram RV, Fink EC, Schneider RK, Al-Shahrour F, Peña P, Breyfogle LJ, Hartwell KA, McConkey ME, Cowley GS, Root DE, Kharas MG, Mullally A, Ebert BL (2014). Csnk1a1 inhibition has p53-dependent therapeutic efficacy in acute myeloid leukemia. J Exp Med.

[74] Chen L, Li C, Pan Y, Chen J (2005). Regulation of p53-MDMX interaction by casein kinase 1 alpha. Mol Cell Biol.

[75] Venerando A, Marin O, Cozza G, Bustos VH, Sarno S, Pinna LA (2010). Isoform specific phosphorylation of p53 by protein kinase CK1. Cell Mol Life Sci.

[76] Inuzuka H, Tseng A, Gao D, Zhai B, Zhang Q, Shaik S, Wan L, Ang XL, Mock C, Yin H, Stommel JM, Gygi S, Lahav G, Asara J, Xiao Z-XJ, Kaelin WG, Harper JW, Wei W (2010). Phosphorylation by casein kinase I promotes the turnover of the Mdm2 oncoprotein via the SCF(beta-TRCP) ubiquitin ligase. Cancer Cell.

[77] Zhang P, Bill K, Liu J, Young E, Peng T, Bolshakov S, Hoffman A, Song Y, Demicco EG, Terrada DL, Creighton CJ, Anderson ML, Lazar AJ, Calin GG, Pollock RE, Lev D (2012). MiR-155 is a liposarcoma oncogene that targets casein kinase-1α and enhances β-catenin signaling. Cancer Res.

[78] Lee C, Etchegaray JP, Cagampang FR, Loudon AS, Reppert SM (2001). Posttranslational mechanisms regulate the mammalian circadian clock. Cell.

[79] Yong TJ, Gan YY, Toh BH, Sentry JW (2000). Human CKIalpha(L) and CKIalpha(S) are encoded by both 2.4- and 4. 2-kb transcripts, the longer containing multiple RNA-destablising elements. Biochim Biophys Acta.

[80] Green CL, Bennett GS (1998). Identification of four alternatively spliced isoforms of chicken casein kinase I alpha that are all expressed in diverse cell types. Gene.

[81] Fu Z, Green CL, Bennett GS (1999). Relationship between casein kinase I isoforms and a neurofilament-associated kinase. J Neurochem.

[82] Budini M, Jacob G, Jedlicki A, Pérez C, Allende CC, Allende JE (2009). Autophosphorylation of carboxy-terminal residues inhibits the activity of protein kinase CK1alpha. J Cell Biochem.

[83] Cegielska A, Gietzen KF, Rivers A, Virshup DM (1998). Autoinhibition of casein kinase I epsilon (CKI epsilon) is relieved by protein phosphatases and limited proteolysis. J Biol Chem.

[84] Graves PR, Roach PJ (1995). Role of COOH-terminal phosphorylation in the regulation of casein kinase I delta. J Biol Chem.

[85] Longenecker KL, Roach PJ, Hurley TD (1998). Crystallographic studies of casein kinase I delta toward a structural understanding of auto-inhibition. Acta Crystallogr D Biol Crystallogr.

[86] Thorne CA, Hanson AJ, Schneider J, Tahinci E, Orton D, Cselenyi CS, Jernigan KK, Meyers KC, Hang BI, Waterson AG, Kim K, Melancon B, Ghidu VP, Sulikowski GA, LaFleur B, Salic A, Lee LA, Miller DM, Lee E, Miller DM (2010). Small-molecule inhibition of Wnt signaling through activation of casein kinase 1α. Nat Chem Biol.

[87] Maritzen T, Lohler J, Deppert W, Knippschild U (2003). Casein kinase I delta (CKIdelta) is involved in lymphocyte physiology. Eur J Cell Biol.

[88] Masuda K, Ono M, Okamoto M, Morikawa W, Otsubo M, Migita T, Tsuneyoshi M, Okuda H, Shuin T, Naito S, Kuwano M (2003). Downregulation of Cap43 gene by von Hippel-Lindau tumor suppressor protein in human renal cancer cells. Int J Cancer.

[89] Brockschmidt C, Hirner H, Huber N, Eismann T, Hillenbrand A, Giamas G, Radunsky B, Ammerpohl O, Bohm B, Henne-Bruns D, Kalthoff H, Leithäuser F, Trauzold A, Knippschild U, Leithäuser F (2008). Anti-apoptotic and growth-stimulatory functions of CK1 delta and epsilon in ductal adenocarcinoma of the pancreas are inhibited by IC261 in vitro and in vivo. Gut.

[90] Knippschild U, Krüger M, Richter J, Xu P, García-Reyes B, Peifer C, Halekotte J, Bakulev V, Bischof J (2014). The CK1 Family: Contribution to Cellular Stress Response and Its Role in Carcinogenesis. Front Oncol.

[91] Sun D, Zhou M, Kowolik CM, Trisal V, Huang Q, Kernstine KH, Lian F, Shen B (2011). Differential expression patterns of capping protein, protein phosphatase 1, and casein kinase 1 may serve as diagnostic markers for malignant melanoma. Melanoma Res.

[92] Fuja TJ, Lin F, Osann KE, Bryant PJ (2004). Somatic mutations and altered expression of the candidate tumor suppressors CSNK1 epsilon, DLG1, and EDD/hHYD in mammary ductal carcinoma. Cancer Res.

[93] Tsai I-C, Woolf M, Neklason DW, Branford WW, Yost HJ, Burt RW, Virshup DM (2007). Disease-associated casein kinase I delta mutation may promote adenomatous polyps formation via a Wnt/beta-catenin independent mechanism. Int J Cancer.

[94] Gao J, Aksoy BA, Dogrusoz U, Dresdner G, Gross B, Sumer SO, Sun Y, Jacobsen A, Sinha R, Larsson E, Cerami E, Sander C, Schultz N (2013). ntegrative analysis of complex cancer genomics and clinical profiles using the cBioPortal. Sci Signal.

[95] Cerami E, Gao J, Dogrusoz U, Gross BE, Sumer SO, Aksoy BA, Jacobsen A, Byrne CJ, Heuer ML, Larsson E, Antipin Y, Reva B, Goldberg AP, Sander C, Schultz N (2012). The cBio cancer genomics portal: an open platform for exploring multidimensional cancer genomics data. Cancer Discov.

[96] Antonov AV, Krestyaninova M, Knight RA, Rodchenkov I, Melino G, Barlev NA (2014). PPISURV: a novel bioinformatics tool for uncovering the hidden role of specific genes in cancer survival outcome. Oncogene.

[97] Kim SY, Dunn IF, Firestein R, Gupta P, Wardwell L, Repich K, Schinzel AC, Wittner B, Silver SJ, Root DE, Boehm JS, Ramaswamy S, Lander ES, Hahn WC (2010). CK1epsilon is required for breast cancers dependent on beta-catenin activity. PLoS One.

[98] Lin S-H, Lin Y-M, Yeh C-M, Chen C-J, Chen M-W, Hung H-F, Yeh K-T, Yang S-F (2014). Casein kinase 1 epsilon expression predicts poorer prognosis in low T-stage oral cancer patients. Int J Mol Sci.

[99] Cheong JK, Nguyen TH, Wang H, Tan P, Voorhoeve PM, Lee SH, Virshup DM (2011). IC261 induces cell cycle arrest and apoptosis of human cancer cells via CK1δ/ε and Wnt/β-catenin independent inhibition of mitotic spindle formation. Oncogene.

[100] Hirner H, Günes C, Bischof J, Wolff S, Grothey A, Kühl M, Oswald F, Wegwitz F, Bösl MR, Trauzold A, Henne-Bruns D, Peifer C, Leithäuser F, Deppert W, Knippschild U (2012). Impaired CK1 delta activity attenuates SV40-induced cellular transformation in vitro and mouse mammary carcinogenesis in vivo. PLoS One.

[101] Sinnberg T, Menzel M, Ewerth D, Sauer B, Schwarz M, Schaller M, Garbe C, Schittek B (2011). β-Catenin signaling increases during melanoma progression and promotes tumor cell survival and chemoresistance. PLoS One.

[102] Vaid M, Prasad R, Sun Q, Katiyar SK (2011). Silymarin Targets ?-Catenin Signaling in Blocking Migration/Invasion of Human Melanoma Cells. PLoS One.

[103] Chien AJ, Moore EC, Lonsdorf AS, Kulikauskas RM, Rothberg BG, Berger AJ, Major MB, Hwang ST, Rimm DL, Moon RT (2009). Activated Wnt/beta-catenin signaling in melanoma is associated with decreased proliferation in patient tumors and a murine melanoma model. Proc Natl Acad Sci U S A.

[104] Lucero OM, Dawson DW, Moon RT, Chien AJ (2010). A re-evaluation of the “oncogenic” nature of Wnt/beta-catenin signaling in melanoma and other cancers. Curr Oncol Rep.

[105] Chien AJ, Haydu LE, Biechele TL, Kulikauskas RM, Rizos H, Kefford RF, Scolyer RA, Moon RT, Long GV (2014). Targeted BRAF inhibition impacts survival in melanoma patients with high levels of Wnt/β-catenin signaling. PLoS One.

[106] Yu DH, Macdonald J, Liu G, Lee AS, Ly M, Davis T, Ke N, Zhou D, Wong Staal F, Li QX (2008). Pyrvinium targets the unfolded protein response to hypoglycemia and its anti-tumor activity is enhanced by combination therapy. PLoS One.

[107] Deng L, Lei Y, Liu R, Li J, Yuan K, Li Y, Chen Y, Liu Y, Lu Y, Edwards CK, Huang C, Wei Y (2013). Pyrvinium targets autophagy addiction to promote cancer cell death. Cell Death Dis.

[108] Zou F-Y, Xie H-L, Chen Z-C, He C-M, Guan Y-J, Li YJ (2003). [Effect of HLCDG1 gene transfection on growth of lung carcinoma cells]. Ai Zheng.

[109] Pribluda A, Elyada E, Wiener Z, Hamza H, Goldstein RE, Biton M, Burstain I, Morgenstern Y, Brachya G, Billauer H, Biton S, Snir-Alkalay I, Vucic D, Schlereth K, Mernberger M, Stiewe T, Oren M, Alitalo K, Pikarsky E, Ben-Neriah Y (2013). A senescence-inflammatory switch from cancer-inhibitory to cancer-promoting mechanism. Cancer Cell.

[110] Toyoshima M, Howie HL, Imakura M, Walsh RM, Annis JE, Chang AN, Frazier J, Chau BN, Loboda A, Linsley PS, Cleary MA, Park JR, Grandori C (2012). Functional genomics identifies therapeutic targets for MYC-driven cancer. Proc Natl Acad Sci U S A.

[111] Bibian M, Rahaim RJ, Choi JY, Noguchi Y, Schürer S, Chen W, Nakanishi S, Licht K, Rosenberg LH, Li L, Feng Y, Cameron MD, Duckett DR, Cleveland JL, Roush WR (2013). Development of highly selective casein kinase 1δ/1ϵ (CK1δ/ϵ) inhibitors with potent antiproliferative properties. Bioorg Med Chem Lett.

