# Supplementary material for: Biological functions of casein kinase 1 isoforms and putative roles in tumorigenesis
Source: Mol Cancer. 2014 Oct 11;13:231. doi: 10.1186/1476-4598-13-231 (PMC4201705; doi:10.1186/1476-4598-13-231)
Supplement: Supplementary file 1 — Authors’ original file for figure 1 [file 12943_2014_1434_MOESM1_ESM.pdf]

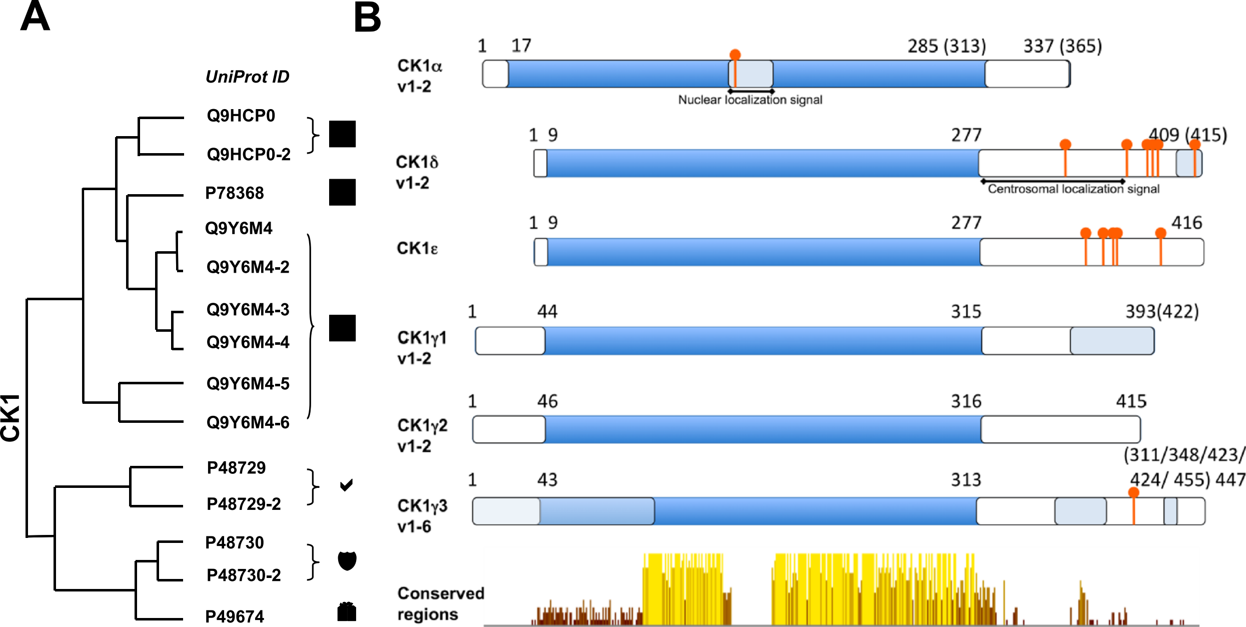
A
B
UniProt ID
Q9HCP0
Q9HCP0-2
P78368
Q9Y6M4
Q9Y6M4-2
Q9Y6M4-3
Q9Y6M4-4
Q9Y6M4-5
Q9Y6M4-6
P48729
P48729-2
P48730
P48730-2
P49674
CK1
CK1α v1-2
1 17 285 (313) 337 (365)
Nuclear localization signal
CK1δ v1-2
1 9 277 409 (415)
Centrosomal localization signal
CK1ε
1 9 277 416
CK1γ1 v1-2
1 44 315 393(422)
CK1γ2 v1-2
1 46 316 415
CK1γ3 v1-6
1 43 313 (311/348/423/ 424/ 455) 447
Conserved regions
