# Supplementary material for: Biological functions of casein kinase 1 isoforms and putative roles in tumorigenesis
Source: Mol Cancer. 2014 Oct 11;13:231. doi: 10.1186/1476-4598-13-231 (PMC4201705; doi:10.1186/1476-4598-13-231)
Supplement: Supplementary file 2 — Authors’ original file for figure 2 [file 12943_2014_1434_MOESM2_ESM.pdf]

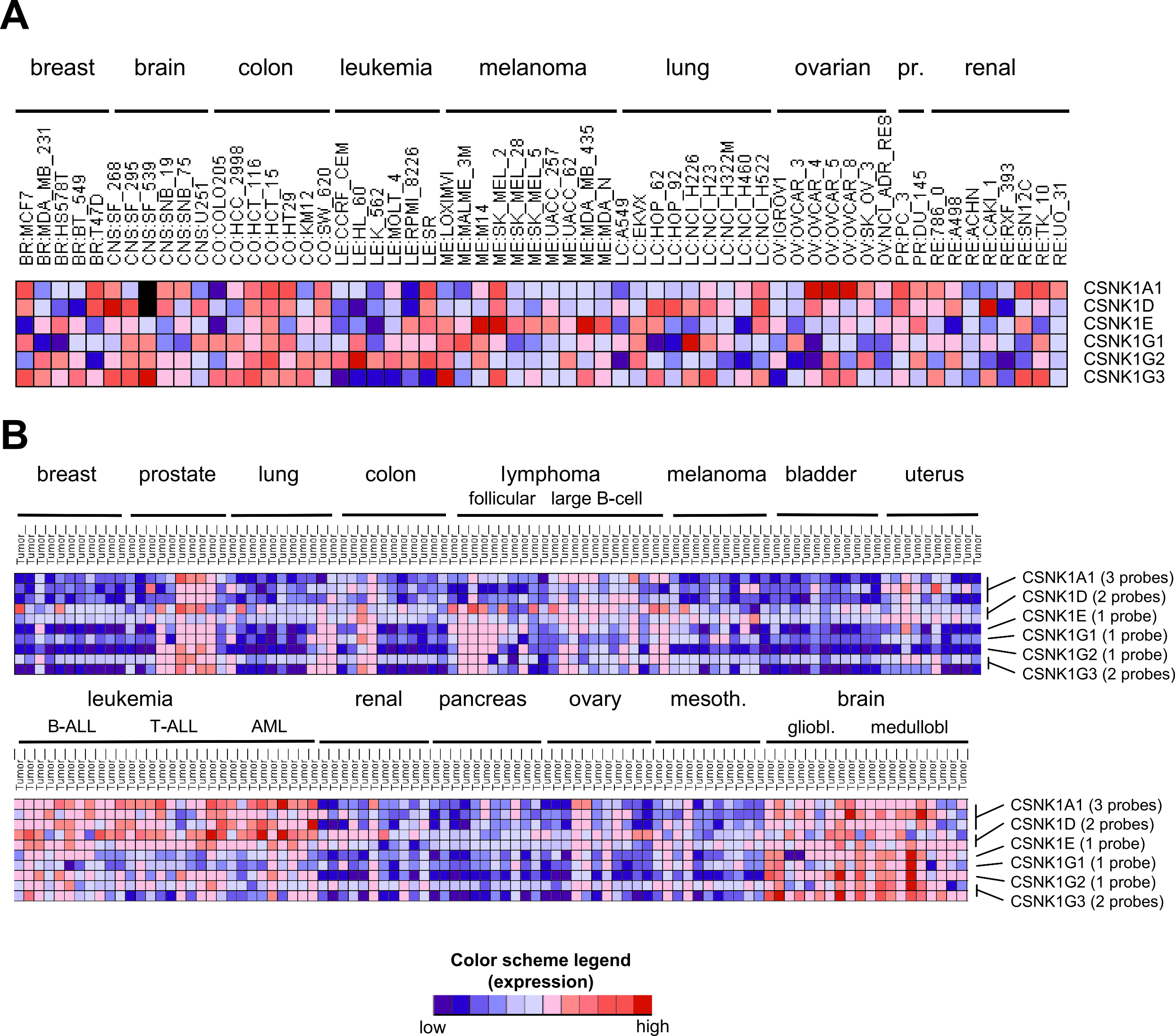
A
breast
brain
colon
leukemia
melanoma
lung
ovarian
pr.
renal
BR:MCF7
BR:MDA_MB_231
BR:HS578T
BR:BT_549
BR:T47D
CNS:SF_268
CNS:SF_295
CNS:SF_539
CNS:SNB_19
CNS:SNB_75
CNS:U251
CO:COLO205
CO:HCC_2998
CO:HCT_116
CO:HCT_15
CO:HT29
CO:KM12
CO:SW_620
LE:CCRF_CEM
LE:HL_60
LE:K_562
LE:MOLT_4
LE:RPMI_8226
LE:SR
ME:LOXIMVI
ME:MALME_3M
ME:M14
ME:SK_MEL_2
ME:SK_MEL_28
ME:SK_MEL_5
ME:UACC_257
ME:UACC_62
ME:MDA_MB_435
ME:MDA_N
LC:A549
LC:EKVX
LC:HOP_62
LC:HOP_92
LC:NCI_H226
LC:NCI_H23
LC:NCI_H322M
LC:NCI_H460
LC:NCI_H522
OV:IGROV1
OV:OVCAR_3
OV:OVCAR_4
OV:OVCAR_5
OV:OVCAR_8
OV:SK_OV_3
OV:NCI_ADR_RES
PR:PC_3
PR:DU_145
RE:786_0
RE:A498
RE:ACHN
RE:CAKI_1
RE:RXF_393
RE:SN12C
RE:TK_10
RE:UO_31
CSNK1A1
CSNK1D
CSNK1E
CSNK1G1
CSNK1G2
CSNK1G3
B
breast
prostate
lung
colon
lymphoma
follicular
large B-cell
melanoma
bladder
uterus
Tumor
CSNK1A1 (3 probes)
CSNK1D (2 probes)
CSNK1E (1 probe)
CSNK1G1 (1 probe)
CSNK1G2 (1 probe)
CSNK1G3 (2 probes)
leukemia
B-ALL
T-ALL
AML
renal
pancreas
ovary
mesoth.
brain
gliobl.
medullobl
Tumor
CSNK1A1 (3 probes)
CSNK1D (2 probes)
CSNK1E (1 probe)
CSNK1G1 (1 probe)
CSNK1G2 (1 probe)
CSNK1G3 (2 probes)
Color scheme legend
(expression)
low
high
