# Supplementary material for: Biological functions of casein kinase 1 isoforms and putative roles in tumorigenesis
Source: Mol Cancer. 2014 Oct 11;13:231. doi: 10.1186/1476-4598-13-231 (PMC4201705; doi:10.1186/1476-4598-13-231)
Supplement: Supplementary file 3 — Authors’ original file for figure 3 [file 12943_2014_1434_MOESM3_ESM.pdf]

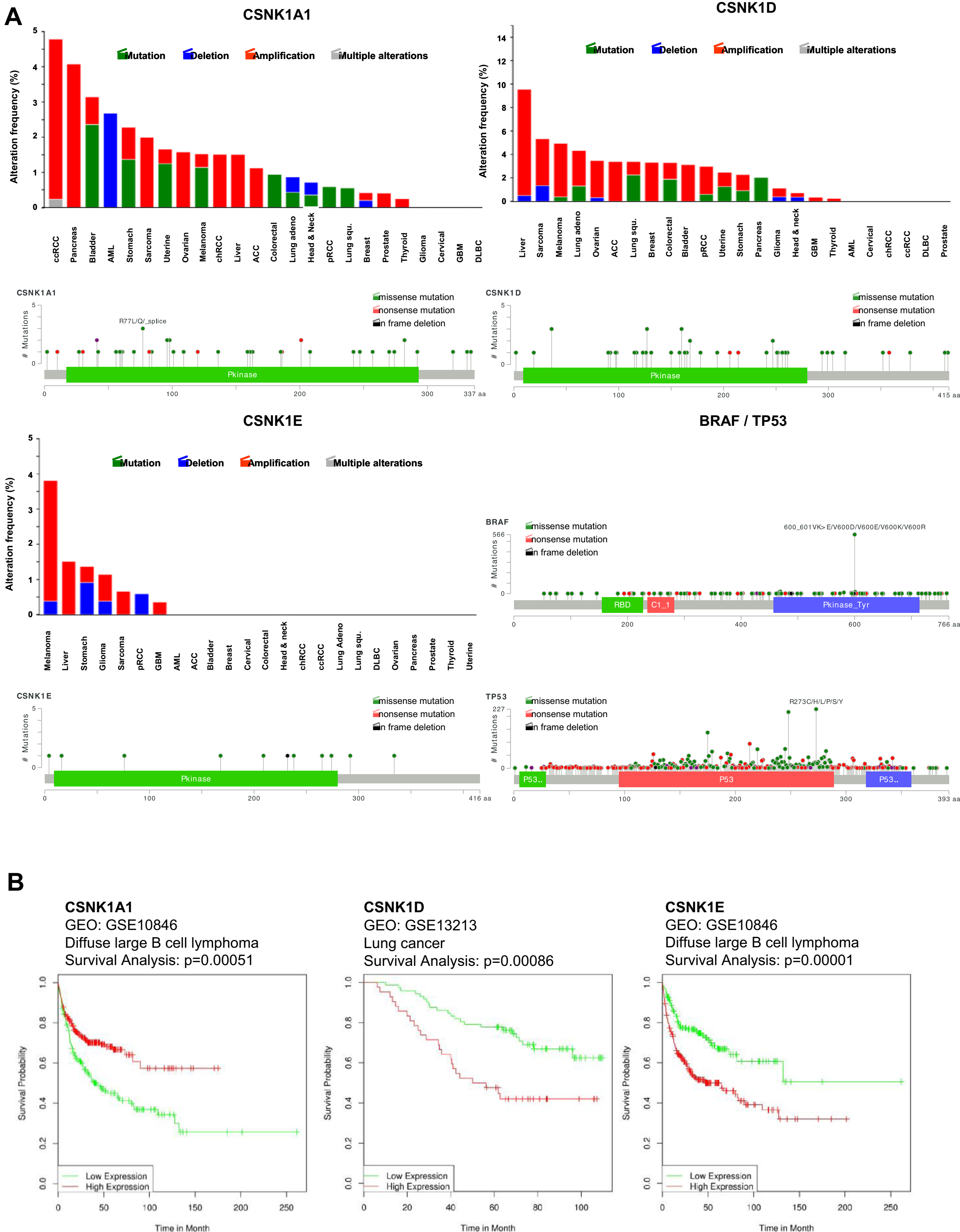
A
CSNK1A1
Mutation
Deletion
Amplification
Multiple alterations
Alteration frequency (%)
ccRCC
Pancreas
Bladder
AML
Stomach
Sarcoma
Uterine
Ovarian
Melanoma
chRCC
Liver
ACC
Colorectal
Lung adeno
Head & Neck
pRCC
Lung squ.
Breast
Prostate
Thyroid
Glioma
Cervical
GBM
DLBC
CSNK1D
Liver
Sarcoma
Melanoma
Lung adeno
Ovarian
ACC
Lung squ.
Breast
Colorectal
Bladder
pRCC
Uterine
Stomach
Pancreas
Glioma
Head & neck
GBM
Thyroid
AML
Cervical
chRCC
ccRCC
DLBC
Prostate
CSNK1A1
missense mutation
nonsense mutation
in frame deletion
Mutations
R77L/Q/_splice
Pkinase
337 aa
CSNK1D
Pkinase
415 aa
CSNK1E
Melanoma
Liver
Stomach
Glioma
Sarcoma
pRCC
GBM
AML
ACC
Bladder
Breast
Cervical
Colorectal
Head & neck
chRCC
ccRCC
Lung Adeno
Lung squ.
DLBC
Ovarian
Pancreas
Prostate
Thyroid
Uterine
BRAF / TP53
BRAF
600_601VK>E/V600D/V600E/V600K/V600R
RBD
C1_1
Pkinase_Tyr
766 aa
CSNK1E
Pkinase
416 aa
TP53
R273C/H/L/P/S/Y
P53..
P53
P53..
393 aa
B
CSNK1A1
GEO: GSE10846
Diffuse large B cell lymphoma
Survival Analysis: p=0.00051
CSNK1D
GEO: GSE13213
Lung cancer
Survival Analysis: p=0.00086
CSNK1E
GEO: GSE10846
Diffuse large B cell lymphoma
Survival Analysis: p=0.00001
Survival Probability
Low Expression
High Expression
Time in Month
